# Integration of clotting and fibrinolysis: central role of platelets and factor XIIIa

**DOI:** 10.1042/BSR20240332

**Published:** 2024-09-23

**Authors:** Irina Patalakh, Olga Revka, Agata Gołaszewska, Natalia Bielicka, Tomasz Misztal

**Affiliations:** 1 Department of Chemistry and Biochemistry of Enzymes, Palladin Institute of Biochemistry of the National Academy of Sciences of Ukraine, Ukraine; 2 Department of General and Experimental Pathology, Medical University of Białystok, 15089 Białystok, Poland; 3 Department of Biopharmacy and Radiopharmacy, Medical University of Bialystok, Poland; 4 Department of Physical Chemistry, Medical University of Bialystok, Poland

**Keywords:** clotting, factor XIIIa, fibrin network, internal fibrinolysis, platelet rich plasma

## Abstract

Purpose: The aim of the present study was to establish the role of platelets and activated factor XIIIa (FXIIIa) in the structuring of the fibrin network as well as to clarify the effect of network compaction on clot lysis.

Methods: Turbidimetry was used for the one-stage clotting test where platelet-free plasma (PFP) is regarded as single factor-deficient plasma (platelets as lacking factor) and autologous platelet-rich plasma (PRP) as deficiency corrected plasma. Structural features of the developed and subsequently lysed fibrin network, formed under static and flow conditions, were visualized by confocal microscopy. Results: Thrombin-initiated plasma clotting revealed changes in the shape of the absorption curve, more pronounced in the presence of platelets. These changes correlate with the transformation of the fibrin scaffold during clot maturing. With the combined action of platelets, thrombin and Ca^2+^, plasma clotting passes through two phases: initial formation of a platelet-fibrin network (first peak in the polymerization curve), and then the compaction of fibrin, driven by FXIIIa (the second peak) which can be further modulate by the contractile action of platelets. These structural changes, mediated by platelets and FXIIIa, have been shown to determine subsequent clot lysis.

Conclusions: Platelet aggregates serve as organizing centers that determine the distribution of fibrin in clot volume. The openwork structure of the platelet-transformed fibrin provides the necessary prerequisites for its timely lysis. The revealed aspects of the interaction of platelets and FXIIIa, which accompanies the maturation of a fibrin clot, may lead to new approaches in the pharmacological correction of disorders associated with both thrombotic episodes and bleeding tendency.

## Introduction

Platelets as a key regulator of hemostasis and thrombosis are responsible for delicate balance of coagulation and fibrinolysis pathways. They form an interface on which thrombin generation, fibrin formation and degradation can be triggered, progressed and terminated. The specific interaction between cell surfaces and plasma-derived zymogens and cofactors propagates the production of thrombin in amounts necessary for the effective polymerization of fibrin. Increased thrombin production can alter the fibrin network (towards more dense structure) and stimulate the contraction of the fibrin clot through activation of blood platelets [1,2] which, in turn, modulates rate of fibrinolysis [3,4].

It is commonly accepted that platelets can change the structure of the fibrin network due to the accumulation on their outer surface of functionally active proteins, both released by cells and captured from plasma [5,6]. Thrombin-initiated formation of a 3D fibrin network and its attachment to the surface of platelets proceeds with the obligatory participation of factor XIIIa. In the blood, factor XIII is found in plasma (plFXIII) and in the cytoplasm of platelets (ptFXIII), with the concentration of ptFXIII (inside platelet secretory granules) being 150 times higher than that of plFXIII [7,8]. However, the initial longitudinal cross-linking of fibrinogen D-domains and the subsequent formation of the fibrin network is mediated by plXIII prelocalized to fibrinogen; while the role of ptXIII is still under debate [7].

Phosphatidylserine (PS)-exposed platelets bound clotting factors from plasma and combine them into tenase and prothrombinase complexes, stimulating thrombin production up to five orders of magnitude [9]. Elevated thrombin translates into the smaller fibril’s diameter and pore size of the fibrin network (resulting in more dense structure) and initiates platelet-mediated retraction [10]. Retraction of the fibrin clot occurs due to factor XIIIa-mediated attachment of cross-linked fibrin strands to the platelet GP IIb/IIIa receptors, causing them to stretch, thereby affecting the diffusion rate of clot-entrapped proteins and the accessibility of their specific binding sites on fibrin [11]. Since the process of clot retraction is accompanied by an increase in the platelet packing density, soluble molecules move through diffusion rather than convection [12]. It was also shown that platelet packing density within the thrombus core region is increased compared with the thrombus shell composed of loosely-attached platelets [13]. Hindered diffusional movement of plasma- and platelet-derived proteins in the consolidated core region increases their retention time in the clot seals, thereby increasing their effective concentration locally [12,13]. Such structural changes in platelet-modified fibrin may be favorable for the binding of major components of the plasmin generation system, such as plasminogen (Pg) and tissue plasminogen activator (t-PA) [14]. Thus, platelets, by controlling the transport and retention of solutes, can play a key role in regulating thrombus growth, maturation, stabilization, and then in its subsequent elimination by fibrinolytic factors. Hence, one may suppose that platelets, by coordinating the spatio-temporal regulatory mechanisms underlying coagulation and fibrinolysis, are involved in changing the local hemostatic balance at different stages of clot development.

Platelet’s procoagulant activity and platelet-dependent resistance to fibrinolysis are well-established for external (therapeutic) clotting/lysis systems where fibrinolysis is triggered by administration of lytic agents after clot formation is complete [3]. Less is known about the participation of platelets in the regulation of clot formation and its subsequent degradation during internal (physiological) lysis. Tutwiler et al. shown that platelet-driven contraction of clots corelates with the ∼4-fold increase in the rate of internal fibrinolysis due to the proximity (in retracted clot) of fibrin fibers to each other and hence in the increase of the t‐PA to fibrin ratio. However, the engagement of FXIII in above phenomenon has not been defined [3].

In contrast with external fibrinolysis, internal fibrinolysis should be initiated almost simultaneously with the accumulation of thrombin, which triggers the formation of fibrin monomers, and then protofibrils and fibrin fibers. Fibrin assembly, in turn, is accompanied by the formation of specific binding sites for Pg and t-PA in the D-domains and αC-domains, which are cryptic in fibrinogen molecules and become exposed in fibrin [15–17]. As a result, fibrin polymerization initiates subsequent fibrinolysis. How platelets can integrate these opposing processes is currently an active area of research with a great clinical and social importance [18].

Until recently, the main tool for studying the functional activity of platelets was agonist-stimulated aggregometry. The current spectrum of platelet research tools includes turbidimetry, which allows visualization of platelet-rich plasma (PRP) clotting using turbidimetric curves [19–22].

Like the turbidimetric curves during polymerization of platelet-poor plasma (PPP), PRP curves have a sigmoidal shape reflecting three stages of transformation of individual fibrinogen molecules into a 3D fibrin network [23]. First, a time lag period, which is necessary for the formation and accumulation of fibrin monomers/double-stranded protofibrils. Then, a rapid increase in turbidity, which is caused by the branching and longitudinal growth of fibrin fibers, as well as their lateral association. And finally, a plateau in turbidity, where the cross-sectional area of the fibers becomes maximal, indicating that the lateral association of protofibrils is complete, therefore, the formation of the fibrillar structure of the fibrin clot is finished [24].

Earlier, in our *ex vivo* model of clotting and subsequent lysis, monitored by turbidimetry, we unexpectedly found changes in the shape of the polymerization curve not previously described. Namely, after the polymerization curve reached a plateau, we found the second wave of its rise, which ended with reaching the second plateau [25]. The detected changes in the shape of the curve showed significant individual variability in max absorbance during PRP clotting, but a stable reproduction of the biphasic rise in turbidity.

Consequently, the present study aims to test our hypothesis that the observed effect may be caused by compaction of the fibrin network – due to cross-linking by FXIIIa and generation of contractile force by activated platelets – as well as to clarify the effect of the assumed compaction on clot lysis. Turbidimetric measurements were supported by confocal analyses of clot structure and fibrin formation and lysis under flow conditions.

## Materials and methods

### Chemicals

Thrombin from human plasma (Sigma, U.S.A.); recombinant tissue plasminogen activator (r-tPA), (Actylise, Boehringer Ingelheim GmbH, Germany); fluorogenic substrate for thrombin BOC-Val-Pro-Arg-AFC (Sigma, U.S.A.); Alexa Fluor 488-labeled human fibrinogen (Thermo Fisher Sci., U.S.A.); transglutaminase/cysteine proteinase inhibitor 4(para)-chloromercuribenzoic acid (PCMB), (Sigma, U.S.A.); Type I collagen (HORM suspension, Takeda, Austria). All other chemicals used were of analytical reagent grade quality and purchased from Sigma-Aldrich (U.S.A.).

### Blood collection and preparation

Blood was obtained from apparently healthy human volunteers (taking no drugs) by venipuncture into acid citrate dextrose (ACD) solution, containing 3.8% of citrate-ions. Donors gave informed consent to the use of their blood samples for the purposes of the present study. Venous blood samples were collected according to the recommendations approved by the local Research Ethics Committee of Palladin Institute of Biochemistry of the National Academy of Sciences of Ukraine (No 3, 30.08.2017) in accordance with the standards set by the Declaration of Helsinki.

Citrated blood was centrifuged at 180×***g*** during 20 min without braking. Supernatant was taken and centrifuged at 350×***g***, 20 min, ambient temperature. Second supernatant, PPP, was aspirated and collected. The remaining 1 ml of plasma with platelet pellet was carefully resuspended and used to prepare PRP after platelet counts were measured. PPP was centrifuged at 10000×***g***, 7 min, for platelet-free plasma (PFP) preparation. Platelet-rich plasma (PRP) with platelet count 50 × 10^3^ platelets/μl (PRP_50_) and 300×10^3^ platelets/μl (PRP_300_) was obtained by diluting the platelet suspension with an appropriate volume of PFP.

### Gel-filtered platelet preparation

Gel-filtered platelets were prepared by PRP filtering through Sepharose-2B column with Tyrode’s buffer, pH 7.35 as eluent. The platelet count was adjusted to 50 × 10^3^/μl and 300 × 10^3^/μl with the same buffer supplemented with 0.1% glucose and 0.35% albumin. Platelet function was tested within 2 h after blood collection. Before testing, platelets aggregability to thrombin (0.1 NIH units/ml) was verified, cells which showed impaired response to thrombin or manifested the spontaneous aggregation were excluded from the experiment.

### Turbidity analysis of plasma clotting and platelet procoagulant activity

Thrombin-initiated clotting was evaluated without non-physiological test reagents (ellagic acid, caoline) by a one-stage clotting test [26] in which PFP is regarded as single factor-deficient plasma, platelets as lacking factor, and autologous PRP as deficiency corrected plasma. Namely, 50 μl of PRP (as a cell sample) was placed in a microtiter plate (Microlon, Greiner Bio-One GmbH, Germany) well, then mixed with 50 μl of 0.05 M Tris-HCl buffer with 0.13 M NaCl, pH 7.4 (TBS), and the reaction was initiated by the addition of 50 μl of freshly prepared mixture of 0.5 NIH U/ml thrombin and 24 mM CaCl_2_ in TBS (hence, final concentrations of thrombin and CaCl_2_ were: 0,166 NIH U/ml and 8 mM, respectively).

In a separate experiment, instead of a thrombin-calcium mixture, a reptilase reagent (Ancistron, Tekhnologiya-Standard, Russia) was used, since reptilase is capable of converting fibrinogen into fibrin, but not FXIII to FXIIIa [27]. In other experiment, PFP or PRP was also clotted without CaCl_2_ by 3.3 NIH U/ml (final concentration) of exogenous thrombin. The higher concentration of thrombin was used to offset the amount of endogenous thrombin normally produced in recalcified plasma during exogenous thrombin-induced clotting.

The coagulation process in the test samples was assessed by turbidimetry, which was monitored at 405 nm every 5 min in a Multiscan 2000 microplate reader (Thermo Electron Corporation, Finland) until a constant maximum absorption was achieved in two parallel samples. PFP and PRP variously affect the baseline absorbance so such absorbance was subtracted as a blank and does not affect assay data.

By plotting optical density (OD units) versus time (min), an OD (*t*) curve was obtained and some kinetic parameters were estimated using the clot waveform analysis (CWA) [28]. The initial and final clotting rates were estimated as the first derivative peaks for the first and second ascending arms in the polymerization curve. The area under the curve (AUC) was calculated as the integral of the OD (*t*) function.

### Thrombin generation measurements

The kinetics of endogenous thrombin generation was recorded during clotting of PFP or PRP in a duplicate using 0.2 μM (final concentration) of thrombin-sensitive fluorogenic substrate BOC-Val-Pro-Arg-AFC (Sigma, U.S.A.) added together with a clotting initiating reagent (mixture of 24 mM CaCl_2_ and 0.5 NIH U/ml of thrombin in TBS). Thrombin generation was estimated as velocity of fluorescence increase (the first derivative, d*F*/d*t*). The fluorescence of 7-amino-4-trifluoromethylcoumarin (AFC) released after cleavage of the substrate with thrombin was monitored at 37°C at 1-min intervals for 90 min using a FL-800 microplate reader (U.S.A.) at an excitation wavelength of 380 nm and an emission wavelength of 480 nm.

### Fibrin monomer-derived clot preparation

Stock fibrin was prepared by mixing 19 ml of 0.25% human fibrinogen with 1 ml of thrombin (100 NIH U/ml) with 0.4 mM PCMB, used to inactivate fibrinogen-associated FXIIIa. After fibrin dissolution in 0.125% acetic acid, the acidic fibrin monomer was obtained as described earlier [29]. It should be noted that after adding to the 0.05 M Tris-HCl buffer, pH 7.4, the fibrin monomer polymerized to form non-crosslinked, soluble fibrin (its subsequent dissolution in 0.125% acetate buffer did not give precipitation). For clot preparation the stock acidic fibrin monomer (20.7 mg/ml) was rapidly diluted in the wells of a multichannel plate to 630 μg/ml (final concentration) using 0.05 M TBS pH 7.4 (with added 0.02 NIH U/ml thrombin and 8 mM CaCl_2_) causing it to spontaneously polymerize into non-crosslinked fibrin.

### Study of clot architecture by fluorescence confocal laser scanning microscopy

For imaging, Alexa Fluor 488-labeled human fibrinogen (Fg-AF488) was added to PRP samples (300 × 10^3^ platelets/μl) to a final concentration of 0.07 mg/ml. If necessary, rt-PA (3 nM, final concentration) was added to the samples before initiating clotting.

After 2-fold dilution of PRP with TBS, a mixture of 24 mM CaCl_2_ and 0.5 NIH U/ml thrombin in a volume of 1/3 of the total sample volume was added to initiate clotting. In some experiments 10 NIH U/ml thrombin was added without calcium.

After stirring for 20 s, 25 μl aliquots were transferred to a microchamber Ibidi µ-Slide VI (Animalab) and incubated in a humid atmosphere at 37°C protected from light. After 15 and 60 min, the clot structure was imaged using fluorescence microscope with confocal imaging system Zeiss Axio Examiner Z.1 (Carl Zeiss Microscopy GmbH, Germany) equipped with W Plan-Apochromat 20×/1.0 water immersion objective lens, and appropriate lasers and filters for the used here fluorophores. Pictures were recorded with Confocal Scanner Unit CSU-X1 (Yokogawa Electric Corporation, Japan) in one focal plane (2D imaging). Images with a 120 × 120 μm field of view were taken at a randomly selected location. At least 3 pictures of different areas of each clot were taken and one representative image is presented.

### Measurements of platelet-dependent fibrin formation and external fibrinolysis under flow conditions

Whole blood samples (without any thrombin inhibitors), were preincubated for 2 min with Fg-AF488 (to visualize fibrin formation) and supplemented with MgCl_2_ (3 mM) and CaCl_2_ (3 mM) just before being perfused through the parallel-plate flow chamber at a shear rate 1000 s^–1^ over the type I collagen (50 μg/ml)-coated microspots until the start of AF488-fibrin formation on the surface of resulting platelet thrombi. Fluorescent fibrin formation was captured by confocal microscope. Then, Hepes buffer (pH 7.45) supplemented with BSA (0.1%), glucose (0.1%) and rt-PA (30 nM) was started to being perfused to initiate external fibrinolysis recorded as a temporal decrease of fibrin-related fluorescent signal.

### Image analysis

The confocal images were analyzed using Image Color Summarizer software (http://mkweb.bcgsc.ca/color-summarizer/). The colors in the image were grouped into three clusters, where the average color represented the structural element of the fibrin network containing Fg-AF488. This approach allowed us to assess the spatial distribution of Fg-AF488 within the clot and differentiate the fibrin-poor zone (cluster 1), the fibrin network (cluster 2), and the fibrin agglomerated zone (cluster 3). The integral fluorescence intensity and area for individual clusters were obtained after image processing with the Image Color Summarizer, and then the specific intensity was calculated for each cluster as the integrated intensity divided by the cluster area.

### Statistical analysis

We analyzed the data using parametric paired *t*-test either the Mann–Whitney *U*-test (since the normality test was failed) or the Wilcoxon’s test (when a small group of dependent data was used). The Kruskal–Wallis test also was used to compare nonparametric data when indicated. Data were presented as medians or as a mean value and standard error of mean, *M* ± SEM. *P*-values of <0.05 were considered statistically significant. Microsoft Excel 2010 and GraphPad Prism 8 software were used for graphical presentation and statistical processing of experimental data.

## Results

### Platelets in the physiologic range affect the clotting pattern of blood plasma

**Figure 1:**
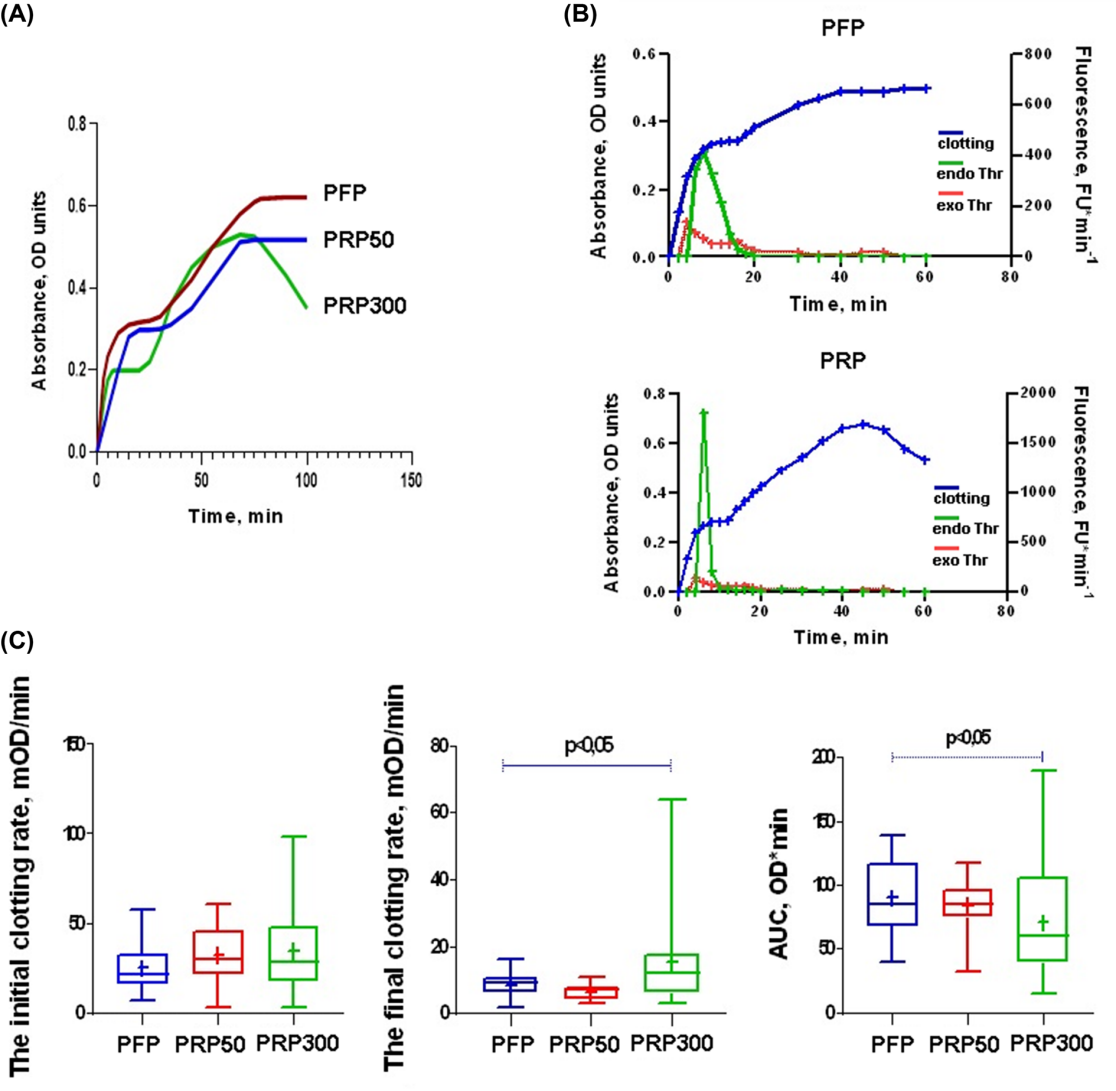
Clotting and thrombin generation in PFP and PRP initiated by mixture of thrombin and CaCl_2_ (**A**) Representative plasma polymerization curves reflected the turbidity changes during thrombin (0.166 NIH U/ml, final conc.) + CaCl_2_ (8 mM, final conc.)-triggered clotting of platelet-free plasma, PFP (blue), platelet-rich plasma with 50 × 10^3^ platelets/μl, PRP_50_ (red) and platelet-rich plasma with 300 × 10^3^ platelets/μl, PRP_300_ (green). (**B**) Time course of clotting (blue) and endogenous thrombin generation (endo Thr, green) in PFP (upper panel) and PRP_300_ (lower panel); exogenous thrombin (0.02 NIH /ml) activity in 0.05 M TBS pH 7.4 (exo Thr, red). Data are from one experiment, representative of three independent experiments. (**C**) Parameters of plasma clotting, *n* = 32, 32 and 14 for PFP, PRP_300_ and PRP_50_, respectively. *P-*values (*<0.05) were determined by Kruskal–Wallis test.

In standard turbidimetric clotting assays, the time to plateau in the absorbance curve is usually used as the end point. Similarly, using turbidimetry to measure thrombin-initiated clotting of recalcified PRP, we observed the appearance of a delayed second wave of turbidity and second plateau in the absorption curve. Using PFP as a cell-free control, we also registered the second absorption wave, which appeared with an even greater delay (Figure 1A).

In general, the change in the shape of the curve became more pronounced in PRP vs. PFP and with an increase in plasma platelet count, in PRP_300_ vs. PRP_50_. These changes were accompanied by an acceleration and increase in the production of endogenous thrombin, which was revealed by an increase in thrombin-mediated cleavage of the fluorogenic substrate by ∼5.5 times compared with the standard one (Figure 1B, bottom panel), while in PFP, an increase in the activity of endogenous thrombin was insignificant (Figure 1B, upper panel). This indicates the elevation of endogenous thrombin production due to the self-enhancing activity provided by exogenous thrombin.

Interestingly, after the stimulation with exogenous thrombin, platelets did not affect the initial clotting rate (Kruskal–Wallis test, *P*=0.122), but significantly influence the final clotting rate (Figure 1C). In addition, for the PRP_300_ samples, the polymerization curve tended to be inverted after reaching maximum turbidity (Figure 1A), which was visually accompanied by a decrease in clot volume, apparently as a result of clot retraction. The platelet counts below the physiological level, as in the samples with PRP_50_, did not cause a decrease in the maximum turbidity, but rather stimulated its increase (Figure 1A). At the same time, the AUC value used to assess the overall changes in clotting kinetics, was significantly reduced only for clots obtained from PRP_300_, which 70.8% of the AUC of clots is obtained using PFP (Figure 1C). This indicates that platelet-mediated reorganization of the fibrin clot structure occurs only when the number of incorporated platelets is within the physiological range.

### Plasma-derived FXIIIa is essential for the second coagulation peak in turbidimetric analysis

**Figure 2:**
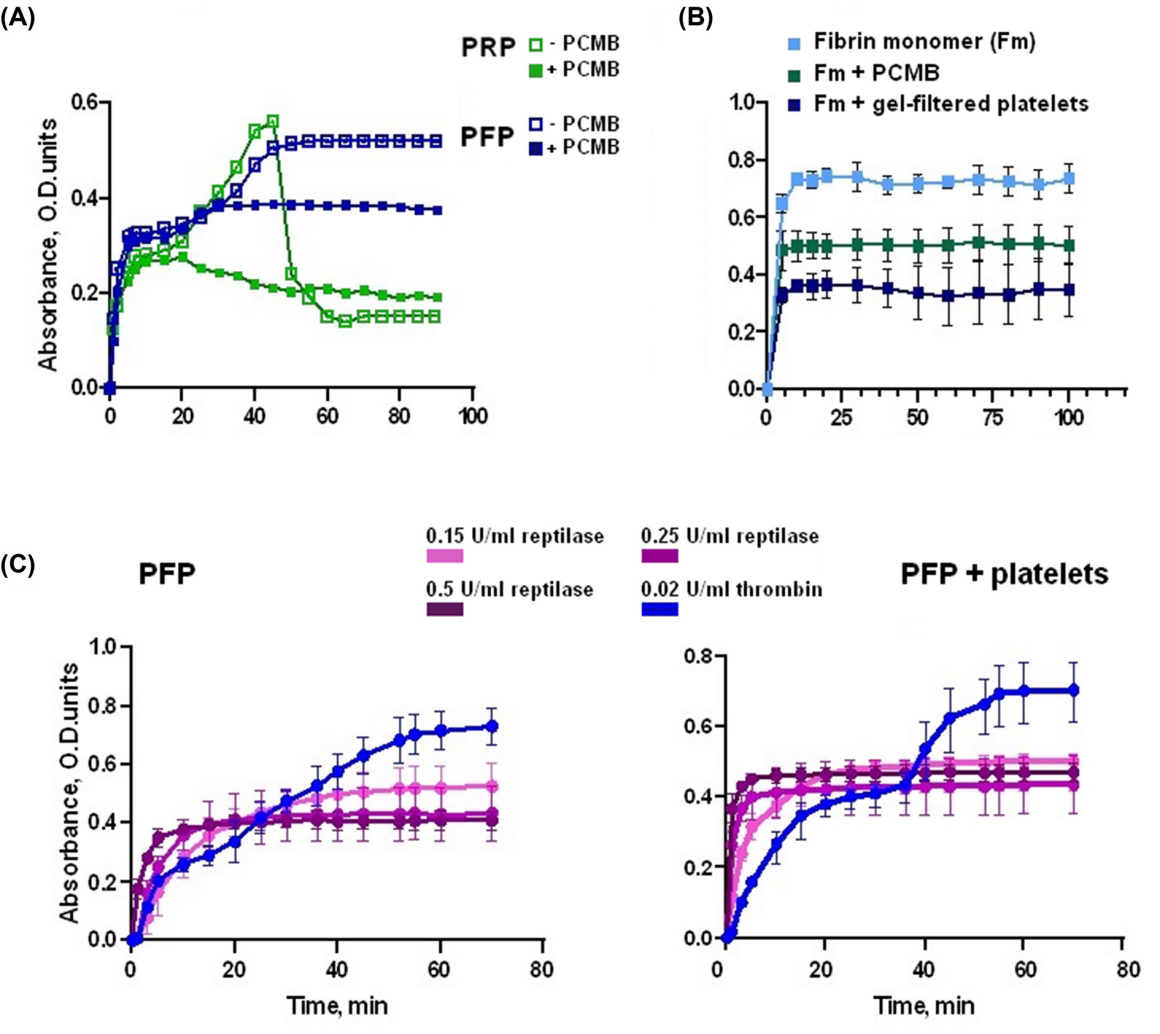
Thrombin-mediated activation of factor XIII affects the late stage of plasma clotting (**A**) Clotting initiated by mixture of thrombin and CaCl_2_ (0.02 NIH units/ml and 8 mM, respectively) preadded in PFP (blue) or PRP_300_ (green): open symbols – non-modified plasma, closed symbols – plasma preincubated during 5 min with PCMB (0.4 mM, final). Plasma pool from three donors. (**B**) The polymerization of: fibrin monomer (light blue), fibrin monomer preincubated for 5 min with 0.6 mM PCMB (green), fibrin monomer with gel-filtered activated platelets (300 × 10^3^ cells/μl) (blue) (*n*=3). (**C**) Clotting of plasma (PFP or PFP + gel-filtered activated platelets, 300 × 10^3^ cells/μl) initiated by reptilase reagent in a final concentration of 0.5 NIH units/ml (dark turquoise), 0.25 NIH units/ml (turquoise), 0.15 NIH units/ml (light turquoise) or 0.02 NIH units/ml thrombin (blue) and 8 mM CaCl_2_. Plasma pool from three donors.

The use of 4(para)-chloromercuribenzoic acid (PCMB), an inhibitor of FXIIIa, resulted in significant suppression of the second absorption wave during PFP clotting and its complete abrogation (together with subsequent retraction) during PRP_300_ clotting (Figure 2A). At the same time, polymerization of the fibrin monomer (which is FXIIIa-independent) also developed without a second absorption wave (Figure 2B), which was not restored even after the addition of gel-filtered thrombin-activated platelets. In contrast, platelets reduced light absorption by almost half, while PCMB partially reversed this platelet-modifying effect on fibrin clot structure.

The use of a reptilase reagent at several concentrations (Figure 2C), instead of a thrombin, clearly confirmed that functionally active thrombin is a prerequisite for two-phase coagulation. Among other functions, it may be necessary for the conversion of FXIII to the active FXIIIa. The addition of platelets, which are capable of secreting activated factor FXIII, did not restore the second peak. This indicates the predominant participation of plasma-derived factor FXIIIa in the development of the observed second phase of clotting.

All these data indirectly indicate the participation of the plasma pool of FXIIIa in the formation of insoluble fibrin during the second phase of plasma clotting.

### Platelet-free plasma forms an isotropic fibrin network during clotting

Using confocal microscopy, we intended to visually detect certain changes in the structure of the fibrin clot that could explain the biphasic shape of the polymerization curve. This study was synchronized with clot turbidity measurements and the same experimental conditions were used. Notably, we compared morphologic changes during plasma clot formation at 15 and 60 min as the time to reach the first and second plateaus, respectively, after the initiation of clotting.

**Figure 3:**
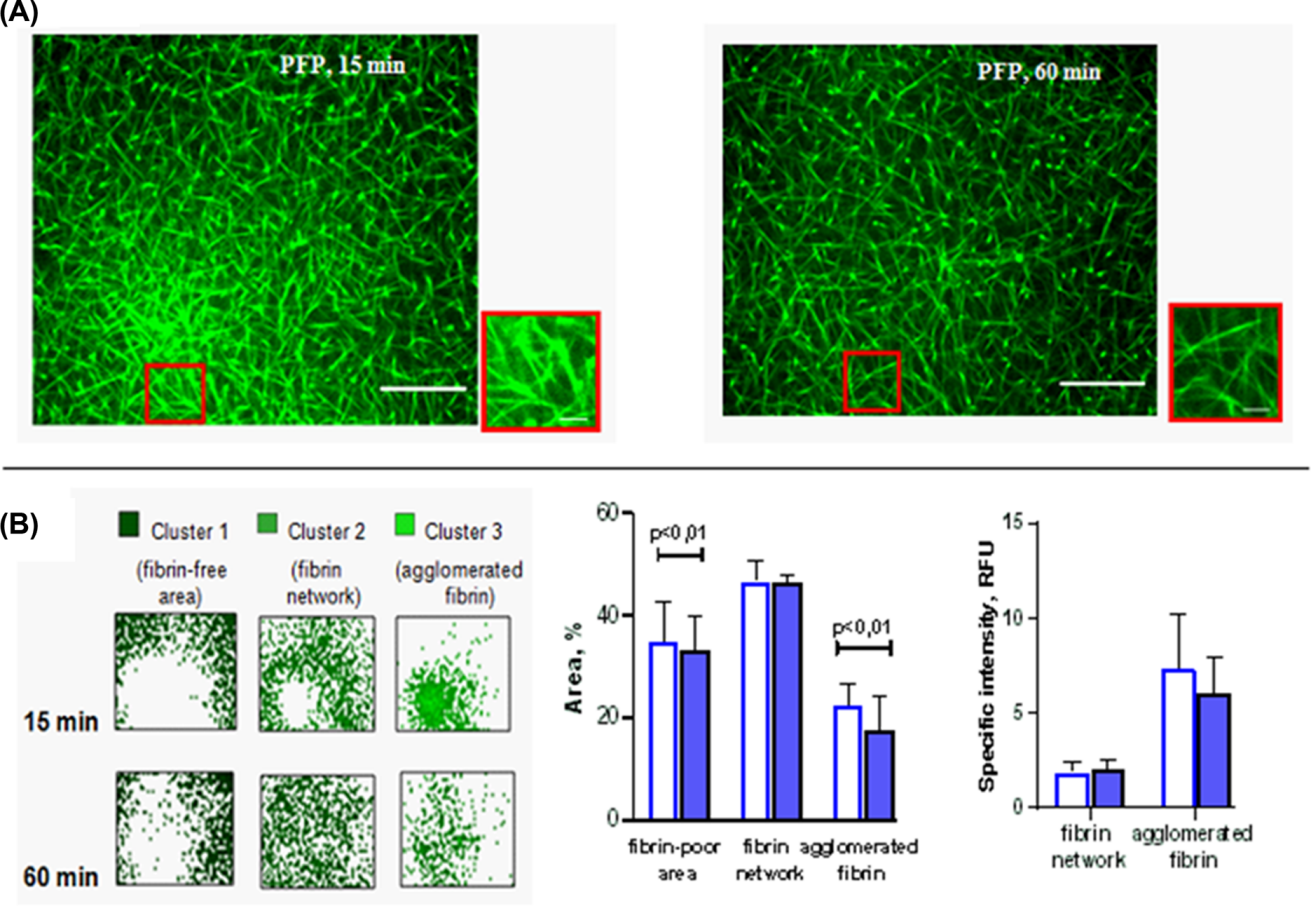
Fibrin network transformation during clotting of PFP (**A**) Images at two time points (15 and 60 min) of fibrin network formation. Tested plasmas were supplemented with fibrinogen-AF488 before clotting initiation by 0.02 NIH units/ml of thrombin with 8 mM CaCl_2_. Images are from one experiment, representative of five independent experiments. Scale bar: 30 μm. Locations of the zoomed images shown in red frame are indicated. Scale bar: 10 μm. Fields in a randomly selected location was chosen. (**B**) The results of confocal microscope data analysis are presented. Image clustering helps to identify three regions in the clot structure: a region not occupied by fibrin (fibrin-poor area), a region of fibrin fibers that form a 3D network (fibrin network), and a region of fibrin deposits (agglomerated fibrin). The total area of the regions depleted of fibrin and occupied by agglomerated fibrin decreased by 60 min (according to the Wilcoxon’s test, the difference is statistically significant, *P*≤0.01, *n*=5), while the total area occupied by the fibrin network region did not change (*P*>0.05). The specific intensity, which reflects the local density of free or associated fibrin, did not change significantly (*P*>0.05).

It was demonstrated that the fibrin network was fully formed at 15 min from the initiation of PFP clotting (Figure 3A), corresponding to the time when the turbidity curve reached the first plateau. The skeleton structure of clots obtained from PFP consisted of straight rod-like fibers organized in a 3D uneven fibrin matrix with a slight agglomeration of fibers. At first, the contours of most fibrin fibers were fuzzy, indicating incomplete lateral association and/or their residual mobility. Then, after 60 min, the fibers became sharper, thinner and connected into a more uniform isotropic network (Figure 3A).

Clustering the color on confocal images into three groups (Figure 3B) helps to reveal the features of the fibrin network and to quantify that over time the structure of a fibrin clot, formed from PFP, becomes more uniform. This is evidenced by the tendency toward a decrease in the area and fibrin density of cluster 3, which displays a group of agglomerated fibers in the image (Figure 3A,B) (*n*=5). The parameters of cluster 2 did not change with time. Together, these events indicate that after 15 min the formation of new fibrin fibers in PFP was insignificant.

### Aggregated platelets regulate the assembly and maturation of the fibrin network over time

**Figure 4:**
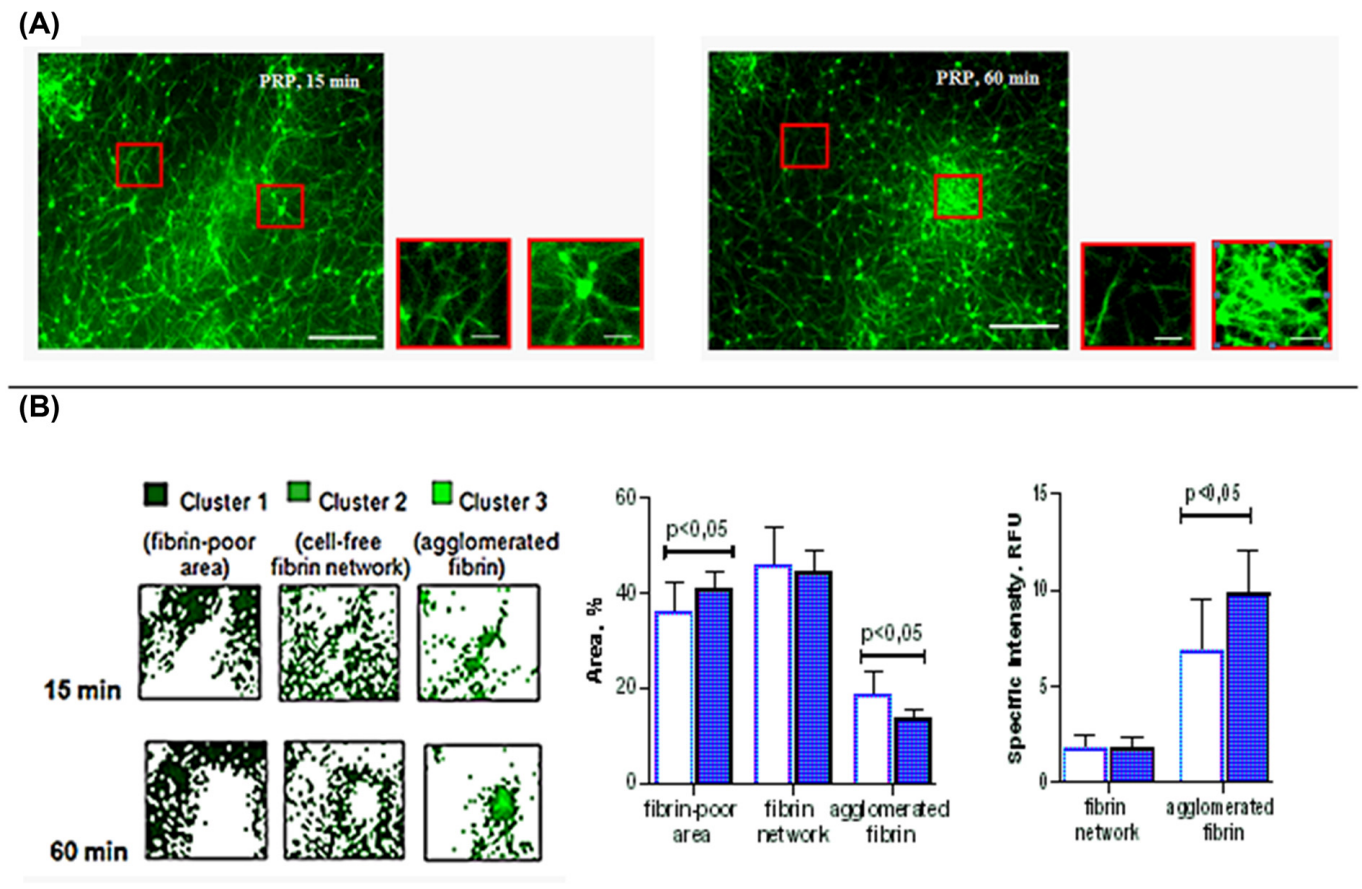
Fibrin network transformation during clotting of PRP (**A**) Images at two time points (15 and 60 min) of fibrin network formation. Tested plasmas were supplemented with fibrinogen-AF488 before clotting initiated by 0.02 NIH units/ml of thrombin with 8 mM CaCl_2_. Images are from one experiment, representative of seven independent experiments. Scale bar: 30 μm. Locations of the zoomed images shown in red frame are indicated. Scale bar: 10 μm. Fields in a randomly selected location was chosen. (**B**) The results of confocal microscope data analysis are presented. Image clustering helps identify three areas in the clot structure: the area not occupied by fibrin (fibrin-poor area), the area of fibrin between platelet aggregates (fibrin network), and the area of fibrin associated with aggregated platelets (agglomerated fibrin). There is no statistically significant difference (*P*>0.05) in total area and specific intensity of cell-free fibrin network between 15 and 60 min but these parameters increased significantly for fibrin-depleted region and agglomerated fibrin (the Wilcoxon’s test, *P*≤0.05, *n*=7).

When autologous PRP was clotted instead of PFP under the same conditions, a significant difference in the clot structure was revealed. Most notably, platelets altered the fibrin matrix, modifying its packaging. If, without platelets, the fibrin network had a uniformly ordered structure formed by interconnected rod-shaped fibers, then it was significantly reorganized by platelets and became heterogeneous (compare Figures 3A and 4A).

First, they formed numerous microaggregates (15 min from the start), which then combined into larger clusters of fibrin and platelets (after 60 min of clot’s maturation). As seen in Figure 4A, each platelet microaggregate is associated with several fibrin fibers radiating outward from its surface. Micro- and then macro-aggregates are combined into star-shaped structures that form a patterned network with nodes and gaps.

The consolidation of fibrin on the surfaces of aggregates caused heterogeneity of its local concentration, which increased at the nodes of the fibrin network and decreased in the gaps. However, further reorganization of fibrin during maturation of a fibrin clot occurred only with the participation of its platelet-associated pattern, while the concentration of fibrin outside platelet aggregates did not change significantly. These visual observations are consistent with quantitative changes in fluorescence intensity. After 60 min, when the turbidity began to rise again after a delay, the specific intensity of cluster 3 increased by 43.0% (Wilcoxon’s test, *P*<0.05, *n*=7), which indicates a more concentrated fibrin in this zone. In addition, after 60 min, the area of cluster 3 contracted by 25.7% (Wilcoxon’s test, *P*≤0.05, *n*=7). Together with an increase in intensity, these changes indicate an increase in fibrin density during agglomeration of platelet microaggregates into larger conglomerates. In addition, a statistically significant increase in the area of the fibrin-free region also indicates some compression of the clot, apparently due to retraction (Figure 4B).

Like PFP, the fluorescence intensity and area of cluster 2 were identical after 15 and 60 min of PRP clotting (Figure 4B) suggesting that the formation of fibrin fibers was completed in the early stages of clot maturation in the presence of platelets. Hence, rise in turbidity after 60 min (Figure 1A) was no longer caused by lateral aggregation of protofibrils, but rather by rearrangement of fibrin fibers into a more compact network with a tendency to decrease the total volume of the clot, which was reflected in an increase in cluster 1 area by 14.6% (Wilcoxon’s test, *P*≤0.01, *n*=7). This result is consistent with a secondary increase in absorption during coagulation of PRP detected by turbidimetry (Figure 1A).

Thus, the maturation of fibrin clots formed from PFP is accompanied by a flattening of the fibrin concentration gradient, while in clots, formed from PRP the structure is complicated due to the alternation of regions with a high and low local fibrin concentration.

### Platelets coordinate progress of fibrinolysis in a spatiotemporal pattern

We hypothesized that platelet-mediated morphological changes in the structure of the clot, which cause local fibrin compaction and probably facilitate molecular transport through the gaps, could be critical for clot degradation efficiency. To verify this assumption, t-PA was preliminarily added to plasma before thrombin/CaCl_2_ mix, to simulate intrinsic fibrinolysis following the clotting of PRP. The t-PA concentration was adjusted so that visible lysis manifested itself later than 15 min, allowing fibrin to first form and then to degrade.

**Figure 5:**
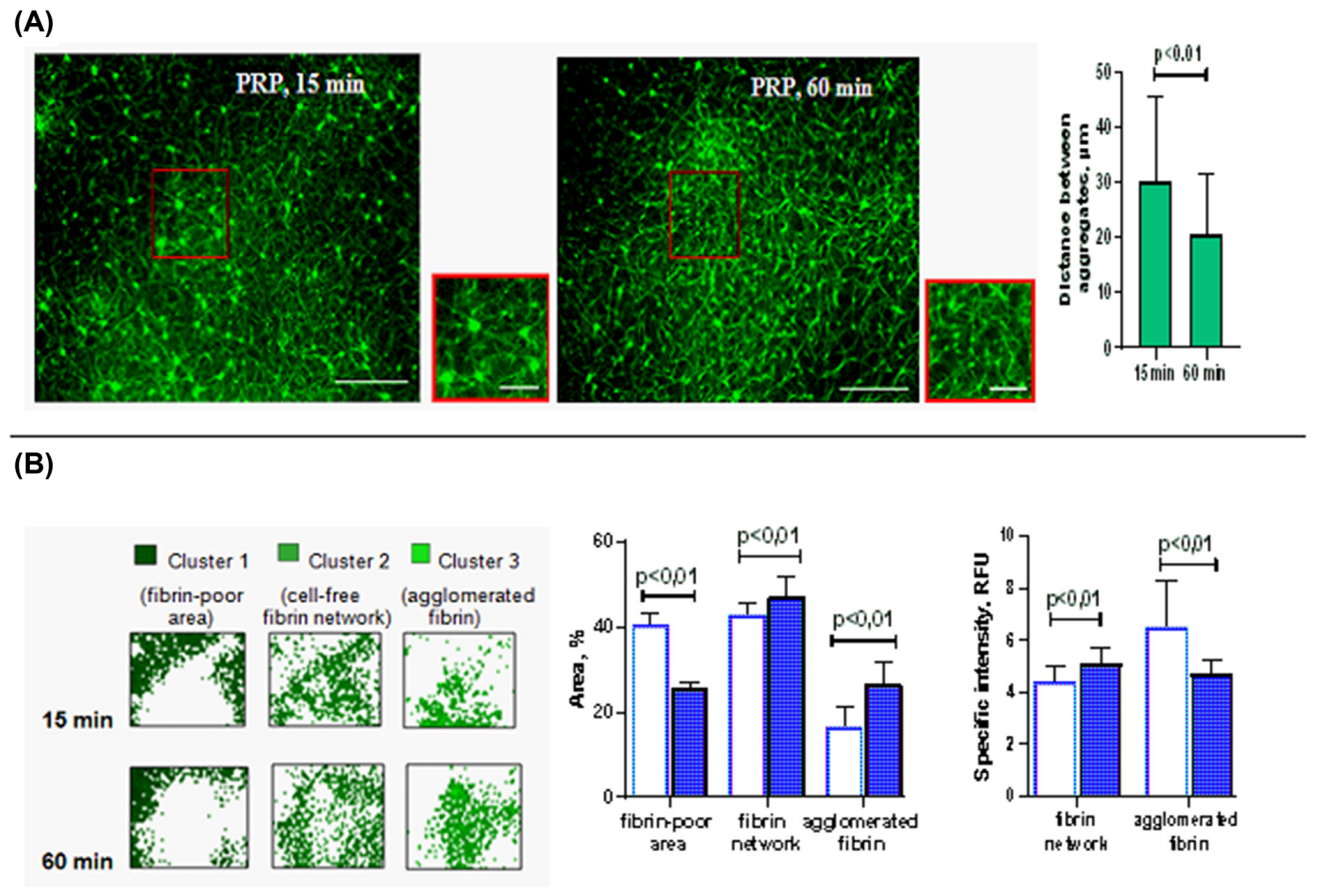
Transformation of the fibrin network during PRP_300_ clotting and clot-initiated internal lysis (**A**) Images at two time points (15 and 60 min) of recombinant tissue-plasminogen activator (rt-Pa)-supplemented fibrin network transformation. Images are from one experiment, representative of three independent experiments. Scale bar: 30 μm. Locations of the zoomed images shown in red frame are indicated. Scale bar: 10 μm. Fields in a randomly selected location was chosen. The distance between cell aggregates was determined in 20 fields randomly selected in the images for each of the three clots. It decreased significantly at 60 min relative to 15 min (Mann–Whitney test, *P*=0.01, *n*=60). (**B**) The results of confocal microscope data analysis are presented. Image clustering helps identify three areas in the clot structure: the area not occupied by fibrin (fibrin-poor area), the area of fibrin between platelet aggregates (fibrin network), and the area of fibrin associated with aggregated platelets (agglomerated fibrin). There was a statistically significant difference in changes in the total area of all three regions and in the specific intensity of cell-free and agglomerated fibrin network between 15 and 60 min (Wilcoxon test, *P*≤0.01, *n*=3).

The principal event during t-PA-mediated degradation of fibrin network (Figure 5) was a disappearance of star-like connections between platelet aggregates still visible at 15 min. As shown in Figure 5A, by the 60th min, internal lysis becomes evident and visualized as a systemic disruption of fibrin fibers radiated from platelet aggregates. By this time, the specific intensity of fluorescence in the region of agglomerated fibrin decreased by 28% (Wilcoxon’s test, *P*≤0.01, *n*=3). Apparently, some of the fibrin associated with cell aggregates was completely dissolved during lysis, giving fibrin degradation products that easily diffused into the platelet-free zone. This is confirmed by a 16% increase (Wilcoxon’s test, *P*≤0.01, *n*=3) in specific intensity in the platelet-free fibrin network. At the same time, the area occupied by agglomerated fibrin increased by 62% (Wilcoxon’s test, *P*≤0.01, *n*=3) (Figure 5B), apparently indicating the disintegration of large platelet aggregates and their dissociation into many microaggregates (Figure 5A, 60 min), which continue to retain fibrin on the surface of platelets and in the intracellular space. In general, it seems that events have turned in the opposite direction to clotting, namely, the local density of fibrin decreased, but the total area that it occupies increased.

It was found that after 60 min of lysis, the clot did not disintegrate into separate fragments, but rather became more compact due to the shortening of fibrin strands between platelet aggregates. As a result, the distance between fibrin-bound cell aggregates decreased at the 60th min by an average of 30% compared with the 15th min (Figure 5A, diagram). This difference, estimated by the Mann–Whitney test, was statistically significant at the *P*=0.01 (*n*=60). These data indicate the ability of the fibrin network to be remodeled during lysis in order to maintain contacts between platelets and the collapsing fibrin scaffold.

### Platelets coordinate the development of fibrin and its lysis under flow conditions

**Figure 6:**
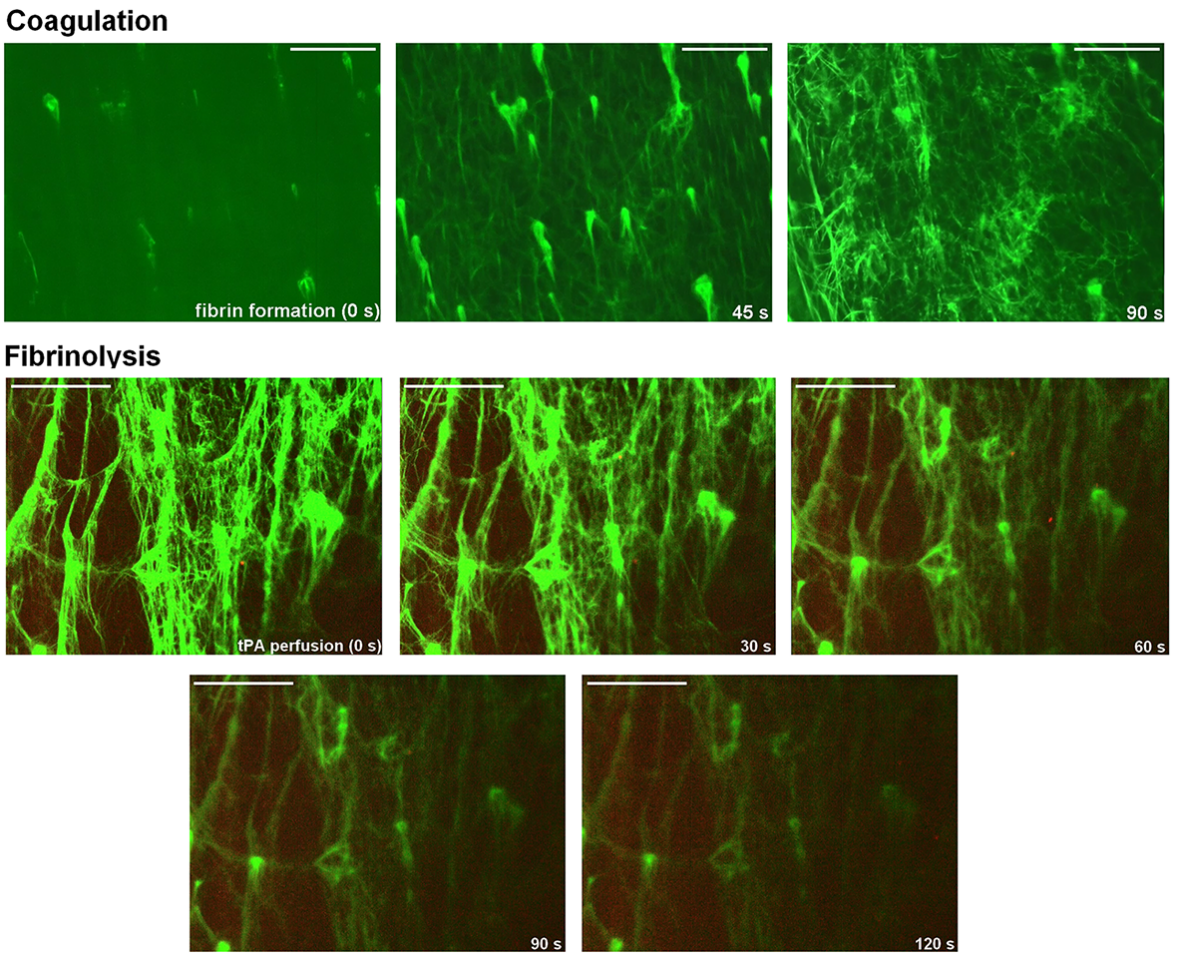
Coagulation and external lysis of fibrin under flow conditions Upper part: whole blood samples without any thrombin inhibitors (supplemented with AF488-fibrinogen) were perfused over the collagen-coated surfaces (shear rate = 1000 s^−1^) to form platelet thrombi which, subsequently, were covered with *in situ*-generated fluorescent fibrin. Progress of fibrin formation and the architecture of fibrin mesh within thrombi was recorded in confocal microscope. Pictures are representative for three independent experiments. Scale bar: 30 μm. Lower part: fibrin clots (formed in a flow chamber under arterial shear rate, i.e. 1000 s^−1^) were washed with a buffer containing recombinant tissue-plasminogen activator (rt-Pa, 60 nM) and sets of confocal pictures were collected to track the decrease of fibrin-related fluorescent signal (reflecting progress of external fibrinolysis, lower part). Representative pictures from one experiment (out of three independent experiments) of fluorescent fibrin at selected time points are presented. ‘*t* = 0’ means fibrin(ogen)-related fluorescence at start point of fibrinolysis. Scale bar: 30 μm.

Similar pattern of clot lysis was observed under flow conditions (arterial shear rate, i.e. 1000 s^−1^), where a rapid fibrin formation (covering platelet aggregates accumulated on collagen-coated thrombotic surface, Figure 6, upper part) was further substituted by a progressive clot ‘melting’, without sudden disruption or fragmentation of clot structure (Figure 6, lower part). Relatively high (30 nM) t-PA was used to visualize dynamics of lysis progress.

### Platelets are unable to coordinate fibrin clot maturation and subsequent lysis under conditions restricting FXIII activity

Considering that fibrinolysis does not require the participation of Ca^2+^- ions, the series of coagulation/lysis experiments was performed in a calcium-free environment to test the role of FXIII in the formation of a fibrin clot structure favorable for physiological lysis. We expected that these conditions would inhibit the stage of FXIII activation and, accordingly, the formation of cross-linked fibrin.

**Figure 7:**
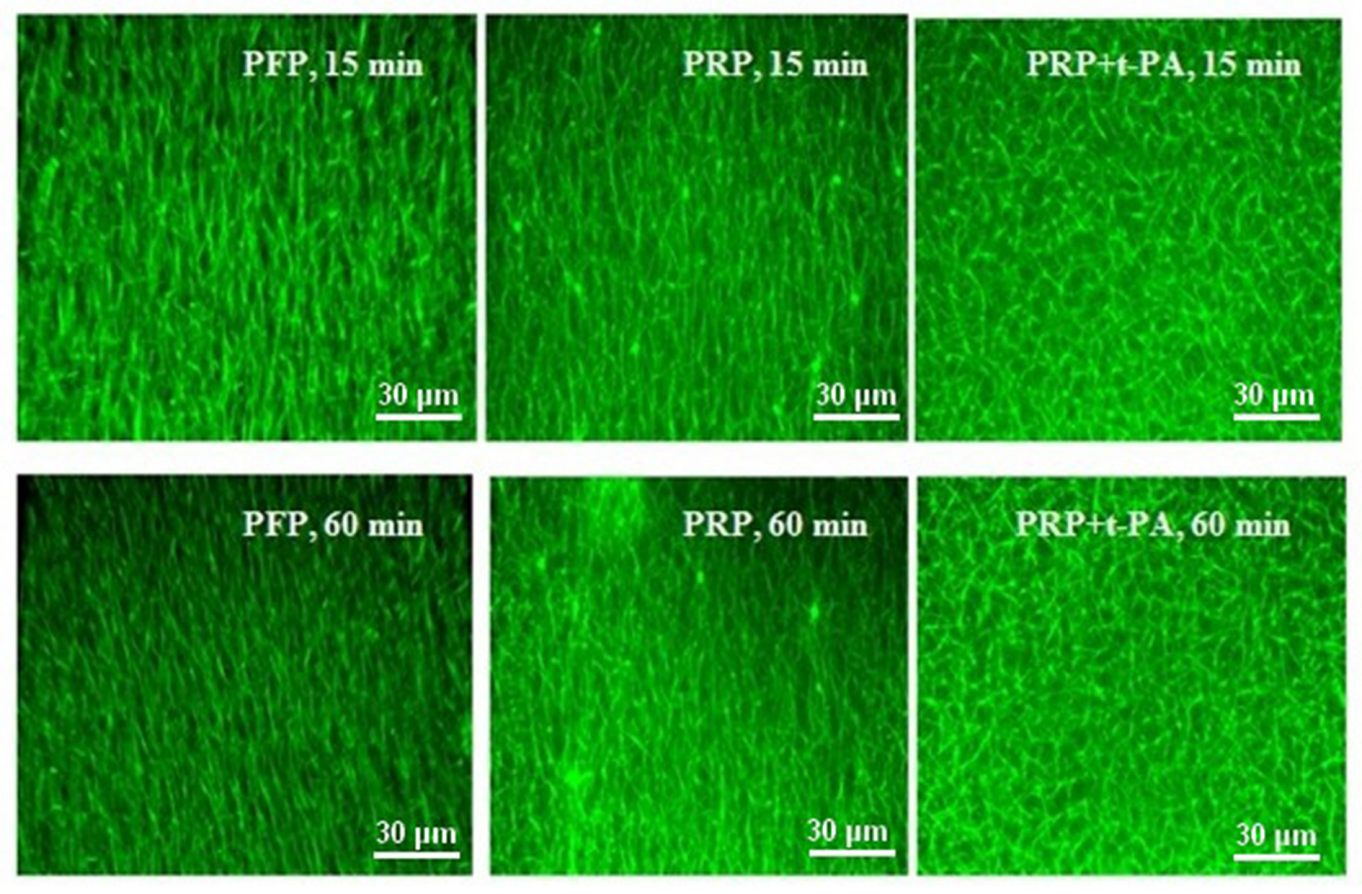
Confocal images of the structure of Ca^2+^-deficient fibrin clots Fibrin clots were formed from Ca^2+^-deficient PFP or Ca^2+^-deficient PRP by thrombin, 10 NIH units/ml. The PRP-derived clot was also partially dissolved by 3 nM rt-PA (final conc.), pre-added to plasma before clotting initiation. Images are from one experiment, representative of three independent experiments. Scale bar: 30 μm.

When PFP or PRP was clotted by relatively high (10 NIH units/ml) of exogenous thrombin without CaCl_2_, unusually long, slightly interconnected fibrin fibers were seen to fill the entire volume of the clot (Figure 7). As follows from a comparison of the upper and lower panels in Figure 7, morphological changes in the clot structure at 15 and 60 min were indistinguishable. This indicates that thrombin (regardless of the presence of platelets) affects only the early stages of fibrin fiber formation and is not able to control the packing of fibers into the network without Ca^2+^. Since the enzymatic activity of thrombin is independent of the presence of calcium ions, Ca^2+^ deficiency can stop FХIIIa-dependent cross-linking of fibrin.

The incorporation of t-PA into the PRP-derived, Ca^2+^-deficient clot during fibrin polymerization, resulted in the shortening of fibrin fibers due to their cross-cutting. However, this did not appear to affect the overall structure of the fibrin network, since other visually distinguishable signs of fibrin clot degradation were not recorded by the 60th min (Figure 7, right column; compare the top and bottom panels). Zones of compaction or rarefaction of fibrin deposits were not found. The specific intensity of fibrin at the 15th and 60th min did not differ statistically (data not shown).

These results confirm the absence of cooperative interactions between the structural elements of the Ca^2+^-deficient fibrin clot during its formation and subsequent lysis, indirectly supporting our hypothesis about the participation of FXIIIa in the orchestration of the clot for lysis at the stage of maturation.

## Discussion

In the present study, we sought to clarify how platelets affect the chain of events that enforce the formation of a fibrin clot, its maturation, and subsequent degradation. By complementing turbidimetric analysis with fluorometric detection of endogenous thrombin, we have shown that platelets can potentiate early events in the coagulation process as well as affect late stages of fibrin polymerization and maturation.

According to our finding, the platelet-driven rise in the polymerization curve starts somewhat earlier than a noticeable amount of thrombin was detected. This could mean that platelets, by driving the coagulation cascade, support generation of the enough thrombin than is required to catalyze fibrin polymerization. This conclusion is consistent with previously reported finding that the normal value of thrombin attained locally in clotting plasma is about 200 nM, while clotting invariably occurs when 10–20 nM of thrombin appears in the plasma, i.e. 5–10% of the possible [1]. By simulating a rapid increase in the plasma thrombin level (due to the addition of exogenous thrombin) we showed that with an excess of thrombin, platelets do not affect the initial clotting rate, but increase the final clotting rate. This might be interpreted that an excessive thrombin can be consumed during the later stages of the coagulation process. Indeed, the manifestation of these events was recorded as an unusual secondary increase in absorption in the late stage of plasma clotting, which we first discovered [25].

The increase in absorption during clotting is known to be associated with а train of successive events: gradual compaction of a fibrin clot due to the lateral association of protofibrils, their assembly into bundles with forming fibers of different diameters, and finally, their spatial packing and factor XIIIa-mediated cross-linking. which leads to the formation of a 3D network of insoluble fibrin. These events develop in the relative time sequence, dominating at certain stages of clot maturation. In this context, we hypothesized that the second phase of absorbance may reflect the action of FXIIIa, when it covalently cross-links fibrin monomers and protofibrils, forming fibrin fibers and creating an optically active 3D network of insoluble fibrin. It seems that during the delay that we observed between the two waves of increased absorption, FXIII was activated by thrombin, which increased the effective concentration of FXIIIa.

It has been proven that the maturation of a fibrin clot first passes through the stage of rapid cross-linking of the γ-chains, and then slow oligomerization of the α-chain [30]. Both stages are served by FXIIIa, which cross-links α-chains more slowly than γ-chains [31]. Based on these data, we suggest that in our model the final coagulation rate may describe the final stage of clot maturation, which depends on the cross-linking of αC-domains by FXIIIa. In our 3-fold diluted plasma clotting model, we were able to provide such a slowdown in coagulation, which helped to unmask the transition from the stage of fiber formation to the stage of its transformation due to cross-linking mediated by FXIIIa.

Our guess about the leading role of FXIIIa in the formation of the second absorption peak is confirmed by the data of other authors. First, it was previously found that thrombin in the presence of fibrin and Ca^2+^ activates FXIII in plasma 40 times slower than it converts fibrinogen into fibrin monomers [32]. The rate-limiting step of FXIII activation is Ca^2+^-induced dissociation of two B subunits, which is critical for full exposure of FXIIIa catalytic activity [33]. It was found that after the addition of FXIII, the initial rate of human fibrinogen polymerization did not change, but the time to reach the maximum absorbance, at a later stage, was significantly increased [34]. Together, these facts help explain the delay we found between the two absorption waves.

Moreover, the kinetics of FXIIIa production is known to be determined by the hitherto generated fibrin, which accelerates the thrombin-induced activation of FXIII by about a hundred time. The removal of B subunit from the parent FXIII molecule during activation is also facilitated by newly formed fibrin [35]. Accordingly, the influence of FXIIIa on the compaction of the fibrin network should appear later, after almost all fibrinogen has been converted to fibrin. This is what we observed from the change in the shape of the polymerization curve, where reaching the first plateau corresponded to the completion of the formation of ‘soft’ fibrin, which should enhance the fibrin-mediated activation of FXIIIa. Activated FXIIIa cross-links fibrin, turning it into insoluble fibrin, thereby increasing clot density [36]. These events can explain the appearance of the second absorption peak in our study.

As an additional argument, thrombin-independent coagulation of PFP (evoked by reptilase) was not accompanied by the formation of a second absorption peak, confirming the critical effect of thrombin on plasma proteins (most notably – FXIII) involved in clot transformation, displayed by this peak.

Finally, we showed that in the presence of PCMB, an inhibitor of FXIIIa, only the first maximum on the PFP and PRP polymerization curves was reproduced, while the second increase in absorption, presumably associated with FXIIIa, was not recorded. This might be also related with the impaired clot retraction, since FXIIIa activity was shown to be vital in the attachment of fibrin to the platelet integrin receptors – a prerequisite for clot contraction [37,38]. Together, all these evidences confirm that platelets in PRP in our experimental model promoted the cross-linking of fibrin by extracellular factor XIIIa, which caused an increase in the final plasma clotting rate.

Confocal microscopy was used to check for a relationship between changes in the turbidity of the coagulated plasma and clot structure. Confocal images, combined with the polymerization curve data, confirm that the first plateau on the curve indicates fibrin scaffold formation, which is completed quite early (in our model, 15 min from the start of clotting under these conditions), and subsequent changes in turbidity are mainly associated with the reorganization of the fibrin network. In particular, the rise of Fg-AF488-signal in PFP and PRP by 60 min corresponds to the second increase in turbidity and indicates the compaction of fibrin during late clotting, which should be caused by the cross-linking of fibrin fibers induced by FXIIIa (in PFP), supported by contractile force generated by activated platelets (in PRP).

Confocal images visualizing the PRP-derived fibrin network in the presence and absence of Ca^2+^ confirmed the crucial role of calcium-ions in clot structure remodeling. Clots formed without Ca^2+^ were covered with a dense ‘brush’ of individual fibrin fibers, which confirms the absence of lateral binding between them. The platelets appear to have been under this fibrous coating, forming a surface for fibrin to attach. Ca^2+^-containing PRP formed clots with a highly ordered openwork structure with nodes of aggregated platelets and spaces between them. Fibrin concentrated in the nodes on platelet aggregates, and also extended beyond them to form rays that connected or intersected in the space between the aggregates, thereby weaving a coarse meshwork. Considering that calcium ions are required for the activation of FXIII [36], we associated the observed changes with the transglutaminase activity of this coagulation factor. These observations are consistent with the report of Mattheij et al., where authors recorded platelet-dependent star-like pattern of fibrin fibers elongation which was FXIII-dependent [39].

Presented here results of the experiments performed under static and flow conditions shown that maturation of the Ca^2+^ – clot develops from numerous microaggregates at the beginning of coagulation to larger conglomerates of fibrin and platelets during the later stages. This leads to the consolidation of fibrin and an increase in its local concentration on the surface of platelet aggregates, while the concentration of fibrin between them remains significantly lower. Based on these results, we believe that platelets and their aggregates serve as organizing centers that control the spatio-temporal distribution of fibrin throughout the clot volume. We think that this form of organization of the fibrin matrix, mediated by platelets with the participation of FXIIIa, facilitates the transport of fibrinolytic proteins to the regions of fibrin accumulation in the volume of the contracted clot, and thereby contributes to its effective degradation.

Based on the above, we hypothesized that the maintenance of cooperative interactions between platelets and the fibrin network is an important criterion for effective fibrinolysis in a disintegrating thrombus. This consideration is supported by morphological changes accompanying the lysis of the platelet-fibrin clots formed in the presence or absence of Ca^2+^. Ca^2+^ was found to be critical for the way in which the lysing clot degrades: Ca^2+^-rich clots shrink during lysis, maintaining cooperativity between aggregates, while Ca^2+^-deficient clots are fragmented uniformly. It seems therefore likely that Ca^2+^, in our model, through the activation of FXIII, is responsible for the tension of fibrin filaments between platelet aggregates. While maintaining continuity and compactness during the lysis, the fibrin network can also provide an effective and secured (protected from inactivation) ‘crawling’ of plasmin along fibrin fibers throughout the entire volume of the degrading clot. According to the study of Samson et al. [40], during lysis of a fibrin clot, the tension and physical arrangement of all connected fibers are continuously redistributed to prevent the decomposing clot from breaking apart. This and other studies [36,37,41]) confirm that cross-linking, which provide a high level of tension on the lysing fibers are critical for accelerating fibrinolysis. Owing to platelets, the clot structure is arranged so that the conflict between packing density and porosity is most effectively resolved. If density determines the stability of the fibrin clot (resistance to premature lysis) when it performs a reparative function, then porosity should be critical for the diffusibility of fibrinolytic factors within the degrading clot. Surprisingly, as we show here, during both the internal (static conditions) and external lysis (flow conditions) the platelet-containing clot does not become more porous or fragmented, but – on the contrary – retains the integrity of its structure, therefore minimizing the tendency of clot fragments detachment (and potential subsequent embolization associated with it).

## Conclusions

It is generally accepted that turbidimetry measures the change in the bulk properties of the clotting plasma and cannot distinguish between the individual processes responsible for the increase in light absorption. Nevertheless, we provide experimental evidence that assessing the shape of turbidimetric fibrin polymerization curves, allows to distinguish between early and late stages of platelet-fibrin clot formation. In the first stage the assembly of the entire fibrin scaffold occurs, and in the last stage the final structure of the clot is formed as a result of its spatio-temporal rearrangement. Similar pattern of events was also observed by the laser confocal microscopy.

Platelets incorporated into the clot structure can alter it in a variety of ways, not only at an early stage, initiating thrombin formation and fibrin polymerization, but also at later stages by participating in FХIII activation. We demonstrated that even a relatively small amount of thrombin is sufficient for a rapid and complete polymerization of fibrinogen in a limited clot volume. Thus, it can be expected that the excess of endogenous thrombin, produced on the surface of activated (procoagulant) platelets, may be required later to promote clot maturation, including FXIIIa-dependent fibers cross-linking, their attachment to the platelet surface, and efficient retraction of the clot. By participating in the formation of the openwork structure of the clot, platelets provide the necessary prerequisites for its full-fledged timely lysis. Namely, platelets, as regulators of cooperative interactions between the elements of the clot are able to control the distance between the aggregates and thereby prevent early fragmentation of the lysing clot. The cooperativity and coordination of structural changes imparts to the collapsing clot the ability to ‘melt’ rather than fall apart into occlusive fragments. This can be vital at the level of the organism as a whole, since it prevents the blockage of small vessels by floating fragments of the lysing clot.

**Figure 8:**
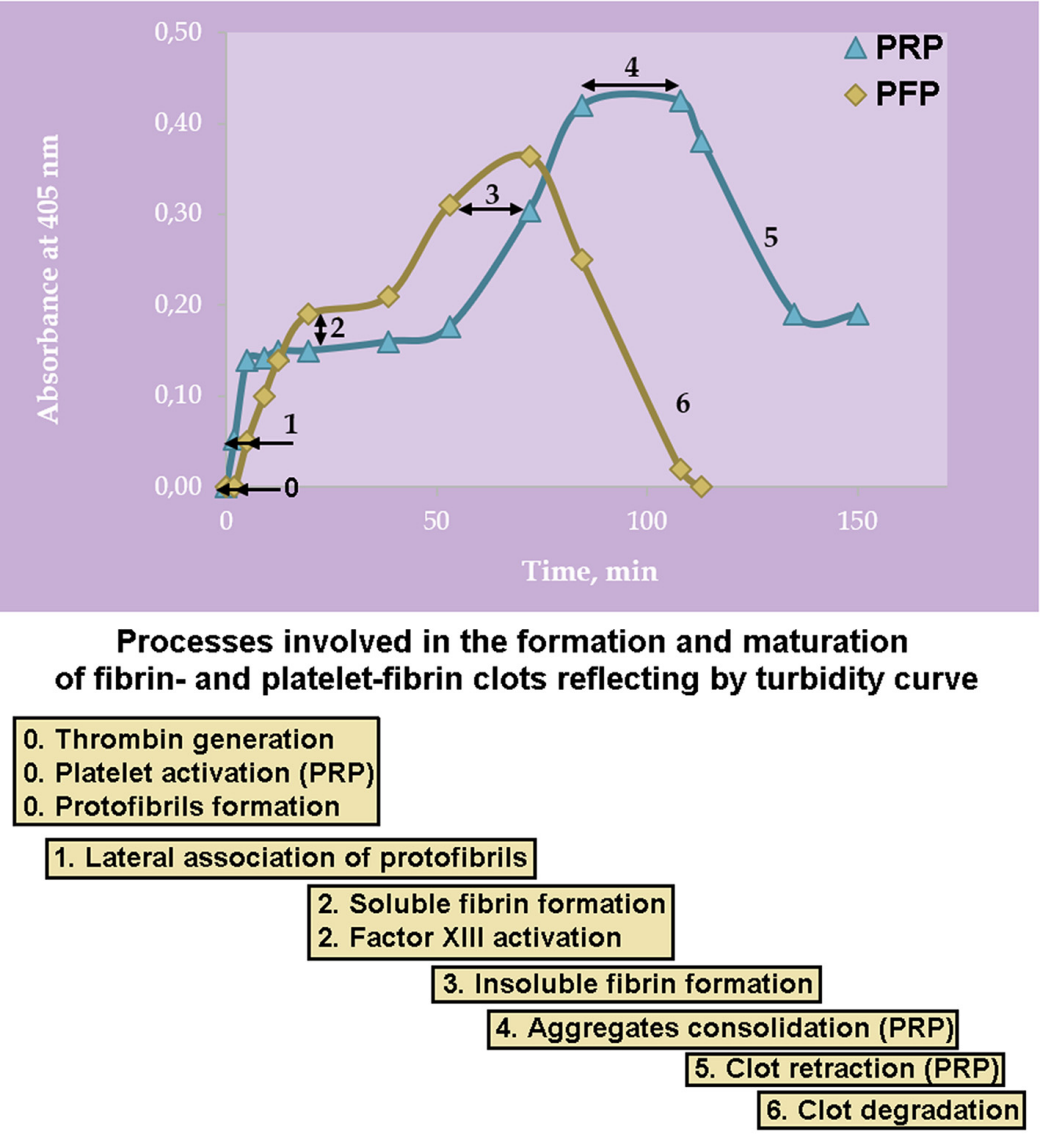
Model of overlapping stages of clot formation and its maturation during turbidimetric measurement

To sum up, our research has shown that the involvement of platelets in clot formation and its development – including lysis – is complex and changes over time. By regulating multiple processes, each of which acts on a different timescale, platelets come into play as procoagulants, and end it as profibrinolytics. Taking into account the limitations of turbidimetric method, in light of the hereby presented results we propose however that commonly accessible turbidimetric assays might be useful in distinguishing and studying the role of platelets in the procoagulatory-profibrinolytic balance (Figure 8).

## Data Availability

The data supporting the findings of this study are available on request from the corresponding author (T.M. and I.P.).

## Abbreviations

AUC: area under the curve
CWA: clot waveform analysis
PCMB: 4(para)-chloromercuribenzoic acid
PFP: platelet-free plasma
PRP: platelet-rich plasma
PS: phosphatidylserine
